# Meta-analysis of skewed data: Combining results reported on log-transformed or raw scales

**DOI:** 10.1002/sim.3427

**Published:** 2008-12-20

**Authors:** Julian P T Higgins, Ian R White, Judith Anzures-Cabrera

**Affiliations:** MRC Biostatistics Unit, Institute of Public HealthRobinson Way, Cambridge CB2 0SR, U.K.

**Keywords:** meta-analysis, logarithm, transformations

## Abstract

When literature-based meta-analyses involve outcomes with skewed distributions, the best available data can sometimes be a mixture of results presented on the raw scale and results presented on the logarithmic scale. We review and develop methods for transforming between these results for two-group studies, such as clinical trials and prospective or cross-sectional epidemiological studies. These allow meta-analyses to be conducted using all studies and on a common scale. The methods can also be used to produce a meta-analysis of ratios of geometric means when skewed data are reported on the raw scale for every study. We compare three methods, two of which have alternative standard error formulae, in an application and in a series of simulation studies. We conclude that an approach based on a log-normal assumption for the raw data is reasonably robust to different true distributions; and we provide new standard error approximations for this method. An assumption can be made that the standard deviations in the two groups are equal. This increases precision of the estimates, but if incorrect can lead to very misleading results. Copyright © 2008 John Wiley & Sons, Ltd.

## INTRODUCTION

Meta-analyses of clinical trials, epidemiological studies and other types of study may involve continuous outcome data. Continuous data can be skewed, typical examples being concentrations (e.g. of plasma triglycerides), other ratio or reciprocal measures (e.g. percentage reduction), measures related to resource use (e.g. recovery time) or assessment scales when there is a large proportion of ‘normal’ participants with scores towards one extreme of the scale (e.g. measures of cognition in population-based studies). Standard inferences on the means of skewed data are valid for large sample sizes due to the central limit theorem, which determines that the mean of the outcome measurements is approximately normally distributed with standard deviation given by the standard error (obtained by dividing the standard deviation by the square-root of the sample size). Since standard meta-analytic methods assume normality in the distribution of the means, but not the raw data [1], they are valid when sample sizes within individual studies are sufficient to enable the central limit theorem to hold approximately.

Focussing on the raw outcome measurements is problematic when the sample size is small, as the standard deviation and mean are affected by extreme values in one direction. It may also lead to loss of efficiency regardless of the sample size. This is well recognized by authors of primary research studies. A common approach to dealing with skewed outcome data is to take a logarithmic transformation of each observation and to conduct the analysis using log-transformed values. This yields, for example, a mean of the log-concentration levels together with a standard deviation of the log-concentration levels, leading directly to a confidence interval for the mean log-concentration level. The mean of the logs can be readily transformed to a geometric mean along with a confidence interval. A logarithmic transformation can offer further advantages, including a focus of the analysis on clinically more appropriate measures of effect [2].

A practical complication in meta-analyses based on summary data is that some studies may present means and standard deviations on the log scale (or geometric means on the raw scale), while other studies present means and standard deviations on the raw scale. It can be difficult to determine exactly which data have been presented. It may, for example, be unclear whether a mean is an arithmetic mean or a geometric mean. Furthermore, some papers may present inappropriate results, such as the exponential of the standard deviation of log-transformed values. Even when the results have been correctly interpreted; however, there remains the problem of combining results on different scales. Here, we present straightforward and approximate transformations that enable meta-analyses on *either* the raw *or* the log-transformed scale, irrespective of how results are presented. We do assume; however, that the nature of all results extracted from papers is known, and we focus on making inferences concerning the comparison of two groups.

## SOME TYPES OF PRESENTATION OF CONTINUOUS OUTCOME DATA

Consider first a single group; say an intervention or a control group from a clinical trial, or a specific exposure group in an observational epidemiological study. Let *n* be the sample size in this single group. Let 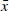 and *s_x_* represent the arithmetic mean and standard deviation of raw (not log-transformed) measurements. Lower and upper limits of a 95 per cent confidence interval for the mean, 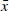 are obtained as


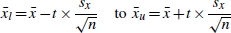


where *t* is the 97.5 percentage point of the *t*-distribution with (*n* − 1) degrees of freedom.

Let 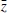 and *s_z_* represent the arithmetic mean and standard deviation of log-transformed measurements. Lower and upper limits of a 95 per cent confidence interval for 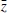 are obtained as


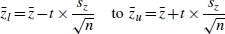


The geometric mean may be obtained as 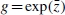. A 95 per cent confidence interval for the geometric mean is given by


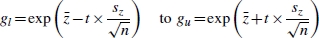


Data available to a meta-analyst might be in one of the following formats, although the list is not exhaustive:

1. Mean and standard deviation of raw measurements (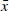 and *s_x_*).
2. Mean and standard error for raw measurements 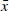 and *s_x_*/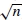).
3. Mean and confidence interval for raw measurements (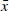, 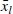 and 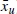).
4. Mean and standard deviation of log-transformed measurements (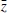 and *s_z_).*
5. Mean and standard error for log-transformed measurements (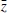 and *s_z_*/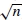).
6. Mean and confidence interval for log-transformed measurements (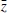, 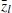 and 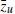).
7. Geometric mean and confidence interval (*g, gl* and *g_u_*).
8. Geometric mean and incorrect standard deviation (*g* and exp(*s_z_*)).

The formulae above can be used to convert any of (1)–(8) to *either*

- the mean (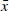) and standard deviation (*s_x_*) for raw measurements *or*
- the mean (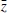) and standard deviation (*s_z_*) for log-transformed measurements.

This should be undertaken before applying the transformation methods below.

## METHODS FOR TRANSFORMING SUMMARY DATA

For variable *X* with a log-normal distribution, such that


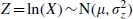


it is a standard result that the mean and variance of *X* are given by


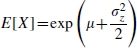


and


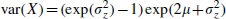


We consider three methods for transforming between log-transformed and raw scales, that is, for estimating the mean and variance of *X* from the sample mean and variance of *Z*, or vice versa. The first two methods exploit the result above. In Method 1, we transform the mean and standard deviation within each group, and then make the comparison across groups. The standard deviations are thus allowed to differ in the two groups. Method 2 follows the same approach as Method 1, but assumes a common standard deviation underlying both groups. This assumption of common standard deviation could be made on either the raw or the log-transformed scale; we choose the latter as a generally more plausible assumption. Method 3 targets the difference between the groups rather than the group means separately. It does not assume a log-normal distribution for the raw data, and is applicable to other transformations as well as the log transformation.

We also derive expressions for the standard errors of the estimators. One possibility for Methods 1 and 2 is to apply standard methods to the converted means and standard deviations for the two groups to obtain a difference in means and its standard error: we call this the ‘*ad hoc*’ approach. However, estimators based on the mean and standard deviation on the log scale are more efficient (have smaller standard errors); hence, the resulting standard errors are too small for conversions from raw to logarithm and too large for conversions from logarithm to raw. We therefore derive alternative standard errors from asymptotic Taylor series approximations. All the estimators below are ‘plug-in’ estimators derived by replacing the population parameters with their estimates; they are therefore likely to be unbiased in large samples but biased in small samples. Further work would be required to remove the small-sample bias.

### Method 1 (separate standard deviations)

An approximate transformation from *Z* to *X* is obtained by substituting estimates for the unknown quantities in the standard result above. Solving the formulae for μ and 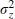 yield the expressions for the opposite conversions. This moment-based approach has been described previously by Whitehead *et al.* [3]. For this method and Method 2, we denote the two exposure (or treatment) groups as *i* = 1 and *i* = 2.

*From raw to logarithm:* To convert 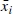 and *s_x,i_* to an approximate mean and standard deviation on the log-transformed scale, take


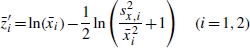


(where the single dash on 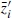 denotes transformation using Method 1), and


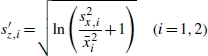


The required difference in means on the log scale from Method 1 is given by


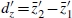


The standard error is given by


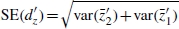


The ‘*ad hoc*’ estimator of 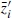 uses the *t*-test formula:


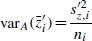


However, this wrongly assumes that 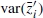 has been computed as an arithmetic mean. The alternative standard error is given by the Taylor series approximation


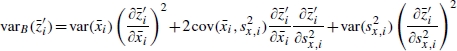


where


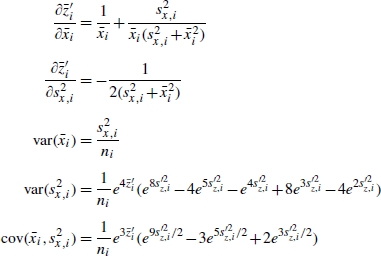


The last two expressions were obtained by approximating 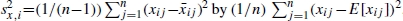 whose asymptotic accuracy was confirmed by simulation. Then we computed


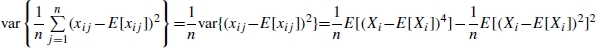


by expanding and using *E[X^n^]* = *E[e^nZ^]* = *e*^*n*μ+*n*^2^σ^2^/2^. A similar argument applies for the covariance.

*From logarithm to raw:* To convert 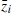 and *s_z,i_* to an approximate mean and standard deviation on the raw scale, take


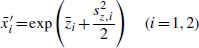


and


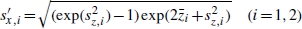


The required difference in means is now


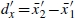


with standard error


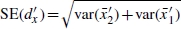


The ‘*ad hoc*’ standard error is estimated using


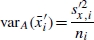


and the alternative standard error by the Taylor series approximation


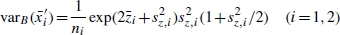


It can be seen that 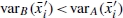, and so the alternative standard error is smaller than the ‘*ad hoc*’ one.

### Method 2 (common standard deviation)

Method 2 is similar to Method 1, but assumes a pooled standard deviation on the log-transformed scale.

*From raw to logarithm:* To convert 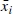 and *s_i_* to an approximate mean and standard deviation on the logarithmic scale, we first transform the standard deviations and then pool them.


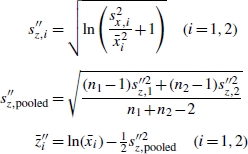


(where the double dash denotes transformation using Method 2). The required difference in means on the logarithmic scale is given by


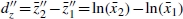


The ‘*ad hoc*’, *t*-test-type, standard error is given by


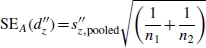


and the standard error, based on Taylor approximation, is given by


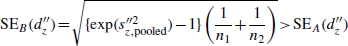


*From logarithm to raw:* To convert 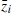 and *s_z,i_* to an approximate mean and standard deviation on the raw scale, we first pool the standard deviations.


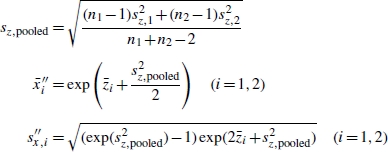


The required difference in means, an ‘*ad hoc*’ standard error and a standard error by Taylor series approximation are given respectively by


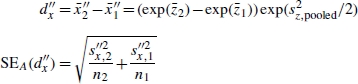


and


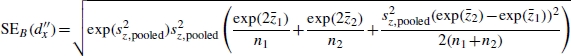


### Method 3

Our third method follows from the following general result and applies directly to the difference between groups rather than the two group means separately. Let *A = g(B)* be the transformation of interest. Then, for example, *g(B)* = ln(*B*) or *g(B)* = exp(*B*) for the current application. Suppose the data have been analysed under a linear model for *B*:


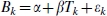


where *T_k_* represents covariates for individual *k*. For the simple comparison of two groups, *T_k_* represents only group allocation, and β is the difference in means. Now let μ_*B*_ be the overall mean, across values of *T*. Then a first-order Taylor series expansion about μ_*B*_ gives


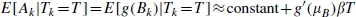


The difference between the means of the two groups can then be estimated, by subtraction, as 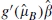. The standard error is obtained similarly as 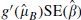. This first-order approximation neglects terms involving β^2^ and beyond, and neglects the term involving the variance of *B*. The former should be acceptable for small effect size β, and the latter if the variance does not depend on *T,* i.e. if the spread of the distribution is similar across groups. The derivatives 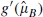 turn out to be the overall geometric mean when transforming from logarithm to raw, and the reciprocal of the overall arithmetic (raw) mean when transforming from raw to logarithm.

*From raw to logarithm:* To convert a difference in means on the raw scale to an approximate difference on the logarithmic scale, take 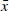 to be the overall arithmetic mean across groups on the raw scale, and use


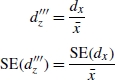


where *d_x_* and SE(*d_x_*) are the difference in means and its standard error from raw means.

*From logarithm to raw:* To convert a difference in means on the logarithmic scale to an approximate difference on the raw scale, take 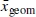 to be the geometric mean of the geometric means across groups (equivalent to the exponential of the arithmetic mean of the means of log-transformed values), and use


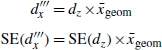


where *d_z_* and SE(*d_z_*) are the difference in means and its standard error from log-transformed values.

## APPLICATION: D9N POLYMORPHISM IN THE LIPOPROTEIN LIPASE GENE AND TRIGLYCERIDE LEVELS

**Figure 1:**
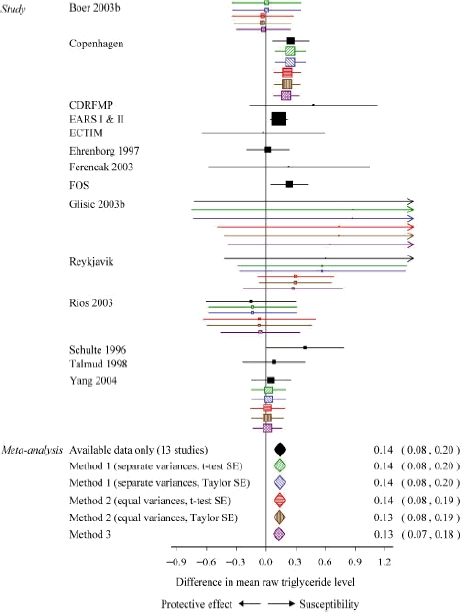
Epidemiological studies of D9N polymorphism in the lipoprotein lipase gene and triglyceride levels: meta-analyses on the raw triglyceride scale, using various conversions from the logarithmic to the raw scale. Where conversions are made, methods are ordered as follows: Method 1 (separate variances, *t*-test SE); Method 1 (separate variances, Taylor SE); Method 2 (equal variances, *t*-test SE); Method 2 (equal variances, Taylor SE); Method 3.

**Figure 2:**
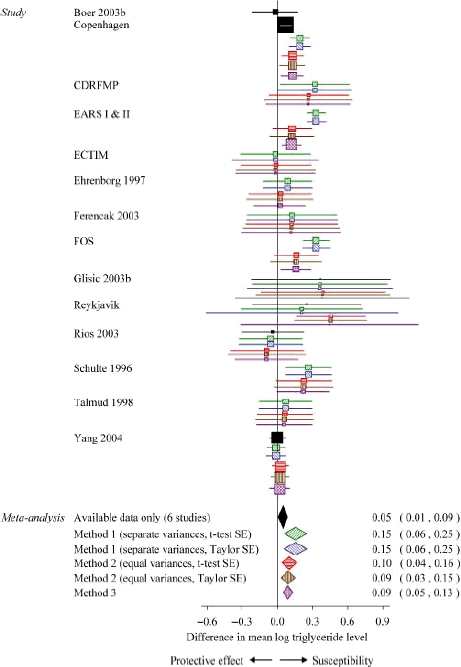
Epidemiological studies of D9N polymorphism in the lipoprotein lipase gene and triglyceride levels: meta-analyses on the log triglyceride scale using various conversions from the raw to the logarithmic scale. Where conversions are made, methods are ordered as follows: Method 1 (separate variances, *t*-test SE); Method 1 (separate variances, Taylor SE); Method 2 (equal variances, *t*-test SE); Method 2 (equal variances, Taylor SE); Method 3.

Sagoo *et al*. conducted a systematic review of association between polymorphisms in the lipoprotein lipase (LPL) gene and coronary heart disease, and also studied plasma levels of cholesterol and triglycerides [4]. We address one particular meta-analysis of 14 studies of the association between triglyceride level and being a carrier or non-carrier of the D9N polymorphism in the LPL gene. Triglyceride levels are typically skewed, and are sometimes presented on the log scale. Through a combination of data extraction from the published reports and correspondence with the original investigators, the review authors obtained means and standard deviations on both logarithmic and raw scales for five studies, on the logarithmic scale only for one study and on the raw scale only for eight studies (Table I). Results for individual studies and meta-analyses are provided in Table II and Figures 1 and 2, for available (‘true’) data and for transformations using our various methods.

**Table I:** Data available for D9N polymorphism in the lipoprotein lipase gene and triglyceride levels.

	Carriers					Non-carriers				
		
		Raw		Log			Raw		Log	
						
	*n*	Mean	SD	Mean	SD	*n*	Mean	SD	Mean	SD
Boer 2003b	34	—	—	0.31	0.58	1002	—	—	0.33	0.53
Copenhagen	241	2.10	1.46	1.05	0.37	8429	1.85	1.54	0.98	0.34
CDRFMP	14	2.05	1.21	—	—	364	1.57	1.11	—	—
EARS I & II	71	1.12	0.34	—	—	1608	0.99	0.80	—	—
ECTIM	22	1.82	1.46	—	—	784	1.84	1.47	—	—
Ehrenborg 1997	15	1.01	0.36	—	—	77	0.99	0.53	—	—
Ferencak 2003	5	2.04	0.92	—	—	195	1.81	0.84	—	—
FOS	58	1.61	0.72	—	—	2200	1.38	1.16	—	—
Glisic 2003b	4	2.42	1.53	0.74	0.60	129	1.64	0.94	0.37	0.49
Reykjavik	10	1.64	1.64	0.20	0.74	274	1.04	0.49	−0.05	0.42
Rios 2003	10	1.60	0.70	0.39	0.41	187	1.75	0.92	0.43	0.50
Schulte 1996	17	1.96	0.82	—	—	644	1.56	0.82	—	—
Talmud 1998	12	1.35	0.52	—	—	96	1.27	0.52	—	—
Yang 2004	235	2.39	1.46	0.74	0.50	1275	2.34	1.26	0.73	0.49

**Table II:** Results of meta-analyses on raw and logarithmic scales, for example, using three transformation methods.

	Available data		Method 1		Method 2		Method 3	
				
	Difference in means	SE	Difference in means	SE	Difference in means	SE	Difference in means	SE
Analysis on raw scale								
Boer 2003b	—	—	0.009	0.175 (0.176)	−0.034	0.145 (0.156)	−0.030	0.138
Copenhagen	0.249	0.096	0.245	0.076 (0.076)	0.213	0.068 (0.070)	0.200	0.067
CDRFMP	0.478	0.329	*0.478*	*0.329*	*0.478*	*0.329*	*0.478*	*0.329*
EARS I & II	0.130	0.045	*0.130*	*0.045*	*0.130*	*0.045*	*0.130*	*0.045*
ECTIM	−0.027	0.315	−*0.027*	*0.315*	−*0.027*	*0.315*	−*0.027*	*0.315*
Ehrenborg 1997	0.020	0.111	*0.020*	*0.111*	*0.020*	*0.111*	*0.020*	*0.111*
Ferencak 2003	0.230	0.414	*0.230*	*0.414*	*0.230*	*0.414*	*0.230*	*0.414*
FOS	0.236	0.097	*0.236*	*0.097*	*0.236*	*0.097*	*0.236*	*0.097*
Glisic 2003b	0.780	0.769	0.877	0.821 (0.829)	0.732	0.588 (0.625)	0.644	0.527
Reykjavik	0.600	0.519	0.567	0.425 (0.435)	0.297	0.186 (0.195)	0.270	0.254
Rios 2003	−0.152	0.232	−0.136	0.228 (0.228)	−0.068	0.270 (0.288)	−0.060	0.203
Schulte 1996	0.393	0.200	*0.393*	*0.200*	*0.393*	*0.200*	*0.393*	*0.200*
Talmud 1998	0.080	0.159	*0.080*	*0.159*	*0.080*	*0.159*	*0.080*	*0.159*
Yang 2004	0.050	0.102	0.027	0.089 (0.090)	0.012	0.082 (0.088)	0.011	0.074
Meta-analysis (random-effects, using Taylor SEs)	0.142	0.031	0.139	0.0310	0.135	0.028	0.125	0.028
	*I*^2^ = 0 per cent; *P*_het_ = 0.57		*I*^2^ = 0 per cent; *P*_het_ = 0.47		*I*^2^ = 0 per cent; *P*_het_ = 0.48		*I*^2^ = 0 per cent; *P*_het_ = 0.48	
Analysis on logarithmic scale								
Boer 2003b	−0.022	0.100	−*0.022*	*0.100*	−*0.022*	*0.100*	−*0.022*	*0.100*
Copenhagen	0.073	0.024	0.192	0.046 (0.041)	0.126	0.054 (0.047)	0.127	0.048
CDRFMP	—	—	0.319	0.159 (0.150)	0.266	0.191 (0.173)	0.264	0.182
EARS I & II	—	—	0.332	0.041 (0.039)	0.123	0.096 (0.085)	0.123	0.042
ECTIM	—	—	−0.017	0.187 (0.153)	−0.015	0.173 (0.152)	−0.015	0.172
Ehrenborg 1997	—	—	0.086	0.108 (0.106)	0.020	0.144 (0.136)	0.020	0.111
Ferencak 2003	—	—	0.125	0.198 (0.194)	0.1250	0.210 (0.199)	0.119	0.215
FOS	—	—	0.335	0.060 (0.058)	0.158	0.111 (0.096)	0.159	0.065
Glisic 2003b	0.370	0.303	0.363	0.316 (0.294)	0.389	0.292 (0.271)	0.384	0.379
Reykjavik	0.250	0.235	0.209	0.419 (0.265)	0.15	0.158 (0.150)	0.388	0.388
Rios 2003	−0.040	0.135	−0.057	0.140 (0.137)	−0.091	0.169 (0.159)	−0.090	0.138
Schulte 1996	—	—	0.266	0.100 (0.099)	0.224	0.128 (0.121)	0.223	0.114
Talmud 1998	—	—	0.071	0.115 (0.114)	0.1	0.125 (0.120)	0.1	0.121
Yang 2004	0.005	0.036	−0.011	0.042 (0.039)	0.021	0.039 (0.037)	0.021	0.043
Meta-analysis (random-effects, using Taylor SEs)	0.047	0.022	0.154	0.050	0.091	0.030	0.091	0.021
	*I*^2^ = 5 per cent; *P*_het_ = 0.38		*I*^2^ = 75 per cent; *P*_het_ < 0.001		*I*^2^ = 15 per cent; *P*_het_ = 0.29		*I*^2^ = 0 per cent; *P*_het_ = 0.49	

*P*_het_ = P-value from a test for heterogeneity among effect estimates in the meta-analysis.

Results in italic are copied across from the available data when no transformations are possible. Main standard errors are calculated using the Taylor approximations; standard errors in parentheses are calculated using the ‘*t*-test’ methods.

Available data on the raw scale allowed meta-analysis of 13 of the studies. We also undertook meta-analyses of all 14 studies, making transformations from the logarithmic to the raw scale wherever this was possible. For five studies, the ‘true’ results can be compared directly with transformations from logarithmic data, and the results are similar in all cases (Figure 1). Furthermore, there are no substantial differences across the different transformation methods (Table II, Figure 1). It is possible for the effect direction to change on transforming between metrics when assuming separate standard deviations. For example, the Boer 2003b transformed to the raw scale using Method 1 (Table II, first row), produces a point estimate that indicates a *higher* mean (by 0.009) in the carriers than in the non-carriers, compared with a *lower* mean (by 0.022) of logs in the observed data. This is because of the larger standard deviation of carriers than non-carriers on the log scale. However, the change in the point estimate is trivial in the context of its confidence interval.

Available data on the log scale allowed meta-analysis of six of the studies. We also undertook meta-analyses of all 14 studies, making transformations from the raw to the logarithmic scale wherever this was possible. Again, for five studies, the ‘true’ and transformed results can be compared directly (Figure 2). One notable discrepancy is in the Copenhagen study, in which the ‘true’ mean difference is smaller than the values estimated by our transformations, and has a somewhat smaller standard error. The bias in the transformations may be because the standard deviations of raw triglyceride levels are relatively large compared with their means, combined with sample size imbalance (see also later simulation results, Table V), or because the data depart more substantially from a log-normal distribution in this study. Point estimates for Method 3 agree well with those for Method 2. In three studies (EARS, FOS and Reykjavik), the assumption of a common standard deviation has a more noticeable effect on the point estimate, so that Method 1 differs from Methods 2 and 3. The studies are also responsible for introducing heterogeneity into the meta-analyses and increasing the summary effect estimate for Method 1. These three studies have substantially different observed standard deviations between carriers and non-carriers (see also later simulation results, Table VI).

**Table IV:** Simulation results for basic set: assumptions hold (log-normal distribution); 100 participants in each group; 10 000 simulations in each set.

		Transformation raw to log scale (μ_*z*, 1_, μ_*z*, 2_) and (σ_*z*, 1_, σ_*z*, 2_)				Transformation log to raw scale (μ_*x*, 1_, μ_*x*, 2_) and (σ_*x*, 1_, σ_*x*, 2_)			
			
		Set 1	Set 2	Set 3	Set 4	Set 1	Set 2	Set 3	Set 4
True means (group 1, group 2)		(0, 0)	(0, 0)	(0, 0.2)	(0, 0.2)	(1.65, 1.65)	(1.02, 1.02)	(1.65, 2.01)	(1.02, 1.25)
True SDs (group 1, group 2)		(1, 1)	(0.2, 0.2)	(1, 1)	(0.2, 0.2)	(2.16, 2.16)	(0.21, 0.21)	(2.16, 2.64)	(0.21, 0.25)
Bias									
Method 1		0.000	0.000	0.002	0.000	0.004	0.000	0.007	0.000
Method 2		0.004	0.000	0.002	0.000	0.004	0.000	0.004	0.000
Method 3		0.004	0.000	0.000	0.000	0.003	0.000	−0.143	−0.004
Standard error									
Method 1	*t*-Test	0.134	0.028	0.134	0.028	0.314	0.029	0.351	0.032
	Taylor	0.446	0.028	0.432	0.028	0.292	0.029	0.326	0.032
	Empirical	0.177	0.028	0.177	0.028	0.293	0.029	0.323	0.032
Method 2	*t*-Test	0.134	0.028	0.134	0.028	0.310	0.029	0.346	0.032
	Taylor	0.172	0.028	0.172	0.028	0.236	0.029	0.264	0.032
	Empirical	0.185	0.029	0.182	0.028	0.235	0.029	0.265	0.032
Method 3	Taylor	0.178	0.029	0.178	0.029	0.142	0.028	0.156	0.031
	Empirical	0.183	0.029	0.179	0.028	0.142	0.028	0.159	0.031
Coverage									
Method 1	*t*-Test	87.2	95.1	86.8	94.6	97.4	94.9	97.4	94.8
	Taylor	99.7	95.1	99.8	94.6	96.1	94.9	96.0	94.8
Method 2	*t*-Test	84.9	94.6	85.3	94.6	99.2	95.3	98.9	95.0
	Taylor	93.2	94.8	93.3	94.8	95.5	95.1	95.2	94.8
Method 3	Taylor	95.3	94.8	95.6	95.3	95.1	95.1	84.1	94.4

Maximum Monte Carlo errors (raw to log) bias: 0.002; *t*-test standard error: 0.0001; Taylor standard error: 0.007; *t*-test coverage: 0.4 per cent; Taylor coverage: 0.3 per cent.

Maximum Monte Carlo errors (log to raw) bias: 0.003; *t*-test standard error: 0.0006; Taylor standard error: 0.0004; *t*-test coverage: 0.2 per cent; Taylor coverage: 0.4 per cent.

**Table V:** Simulation results for sample size imbalance (log-normal distribution); 10 participants in group 1, 100 participants in group 2; 10 000 simulations in each set.

		Transformation raw to log scale (μ_*z*, 1_, μ_*z*, 2_) and (σ_*z*, 1_, σ_*z*, 2_)				Transformation log to raw scale (μ_*x*, 1_, μ_*x*, 2_) and (σ_*x*, 1_, σ_*x*, 2_)			
			
		Set 9	Set 10	Set 11	Set 12	Set 9	Set 10	Set 11	Set 12
True means (group 1, group 2)		(0, 0)	(0, 0)	(0, 0.2)	(0, 0.2)	(1.65, 1.65)	(1.02, 1.02)	(1.65, 2.01)	(1.02, 1.25)
True SDs (group 1, group 2)		(1, 1)	(0.2, 0.2)	(1, 1)	(0.2, 0.2)	(2.16, 2.16)	(0.21, 0.21)	(2.16, 2.64)	(0.21, 0.25)
Bias									
Method 1		−0.051	0.000	−0.051	0.001	−0.115	−0.003	−0.113	−0.002
Method 2		0.072	0.001	0.070	0.002	−0.067	−0.003	−0.069	−0.002
Method 3		0.070	0.001	0.060	0.000	−0.041	−0.003	−0.188	−0.007
Standard error									
Method 1	*t*-Test	0.269	0.064	0.269	0.064	0.878	0.067	0.897	0.069
	Taylor	0.688	0.064	0.676	0.064	0.776	0.067	0.794	0.069
	Empirical	0.356	0.066	0.357	0.067	0.791	0.069	0.800	0.070
Method 2	*t*-Test	0.310	0.066	0.310	0.066	0.759	0.068	0.780	0.070
	Taylor	0.399	0.067	0.398	0.067	0.577	0.068	0.593	0.069
	Empirical	0.401	0.067	0.400	0.067	0.586	0.068	0.602	0.070
Method 3	Taylor	0.341	0.065	0.319	0.060	0.328	0.065	0.363	0.072
	Empirical	0.385	0.067	0.380	0.066	0.349	0.066	0.358	0.068
Coverage									
Method 1	*t*-Test	84.3	92.1	84.1	92.2	91.7	92.3	92.8	92.7
	Taylor	96.2	92.2	95.9	92.2	90.4	92.3	91.7	92.7
Method 2	*t*-Test	86.0	94.5	86.5	94.3	97.7	95.1	97.7	95.0
	Taylor	93.8	94.8	93.6	94.5	93.9	94.8	94.7	94.7
Method 3	Taylor	85.9	92.0	85.1	90.4	92.5	92.4	93.3	94.6

Maximum Monte Carlo errors (raw to log) bias: 0.004; *t*-test standard error: 0.0005; Taylor standard error: 0.009; *t*-test coverage: 0.4 per cent; Taylor coverage: 0.4 per cent.

Maximum Monte Carlo errors (log to raw) bias: 0.008; *t*-test standard error: 0.007; Taylor standard error: 0.009; *t*-test coverage: 0.3 per cent; Taylor coverage: 0.3 per cent.

## SIMULATION STUDY

We undertook a simulation study to compare the methods. Continuous outcome data were simulated for a single, two-group study, according to various distributions, and subjected to the three transformation methods, both converting the raw simulated data to the logarithmic scale and converting the logs of the simulated data to the raw scale. Since we knew the means and standard deviations on both scales (either theoretically or empirically), we could compare the estimated differences in means (and their standard errors) with those that would have been obtained had the data been analysed on the desired scale.

**Figure 3:**
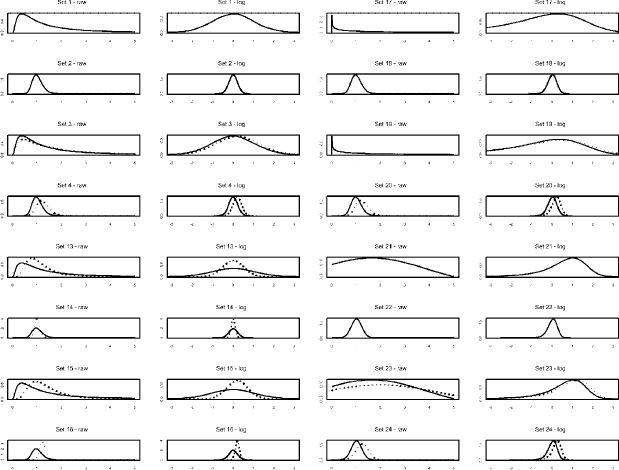
Probability densities of all distributions used in the simulation study. Solid lines represent group 1 and dotted lines group 2.

Our initial set of simulations used log-normally distributed data with equal standard deviations across groups (on the log scale), thus the distributional assumptions underlying all methods hold exactly, and only the asymptotic approximations would affect results. Each group had a sample size of 100. We then evaluated, with further simulations, (i) small sample sizes (10 rather than 100); (ii) imbalance in sample sizes across the two groups; (iii) different standard deviations in the two groups; (iv) a different skewed distribution (gamma distribution); and (v) lack of serious skew (normal distribution, with negative values rejected). The gamma and normal distributions were chosen to have (before rejection of samples) identical means and standard deviations on the raw scale to the initial log-normally distributed data. Full details of the data generation and the parameter values are provided in Table III. Illustrations of all distributions simulated are included in Figure 3.

**Table III:** Data generation and parameter values for all simulations. Parameter values for *Z* in sets 17–24 (in italics) are derived empirically.

					Log scale (Z)				Raw scale (X)			
						
					Group 1		Group 2		Group 1		Group 2	
								
Basic set: assumptions hold (log-normal distribution)	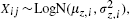	1	100	100	0	1	0	1	1.65	2.16	1.65	2.16
	*Z_ij_* = ln(*X_ij_*), *j* = 1,…, *n_i_*	2	100	100	0	0.2	0	0.2	1.02	0.21	1.02	0.21
	equivalent to 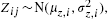	3	100	100	0	1	0.2	1	1.65	2.16	2.01	2.64
	*X_ij_* = exp(*Z_ij_*)	4	100	100	0	0.2	0.2	0.2	0.2	0.21	1.25	0.25
Small sample size	As above	5	10	10	As basic set							
		6	10	10								
		7	10	10								
		8	10	10								
Sample size imbalance	As above	9	10	100	As basic set							
		10	10	100								
		11	10	100								
		12	10	100								
Different standard deviations	As above	13	100	100	0	1	0	0.5	1.65	2.16	1.13	0.60
		14	100	100	0	0.2	0	0.1	1.02	0.21	1.01	0.10
		15	100	100	0	1	0.2	0.5	1.65	2.16	1.38	0.74
		16	100	100	0	0.2	0.2	0.1	0.2	0.21	1.23	0.12
Alternative skew (gamma distribution)	*X_ij_* ∼ Gamma(α_*i*_, λ_*i*_),	17	100	100	−*0.57*	*1.94*	−*0.57*	*1.94*	As basic set			
	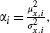	18	100	100	−*0.001*	*0.20*	−*0.001*	*0.20*				
	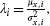	19	100	100	−*0.56*	*1.94*	−*0.38*	*1.94*				
	*Z_ij_* = ln(*X_ij_*), *j* = 1, …, *n_i_*	20	100	100	−*0.0009*	*0.20*	*0.20*	*0.20*				
No skew (normal distribution, positive values only)	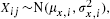	21	100	100	*0.59*	*0.98*	*0.59*	*0.98*	As basic set			
	rejected and replaced if	22	100	100	−*0.002*	*0.21*	−*0.003*	*0.21*				
	*X_ij_*<0 *Z_ij_* = ln(*X_ij_*), *j* = 1, …, *n_i_*	23	100	100	*0.56*	*0.98*	*0.79*	*0.98*				
		24	100	100	−*0.002*	*0.21*	*0.20*	*0.21*				

For each scenario and parameter set (each row in Table III), we undertook 10000 simulations. Each simulation produced three estimates (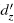, 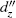 and 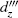) with five standard errors (SE_*A*_(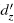), SE_*B*_(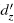), SE_*A*_(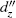), SE_*B*_(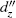) and SE(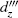)) for transformations from the raw to the log scale, and the corresponding numbers for transformations from the log to the raw scale. We summarized them using measures of bias, precision and coverage as follows, where *d* represents one of the three estimates.

*Bias:* Bias was defined as mean estimated difference in means *(d)* minus true difference in means. For log-normal simulations and gamma simulations (raw scale only), the true values were known theoretically. For the others, the true mean differences were estimated empirically across simulations. We present mean bias for log-normal simulations and median bias for gamma and normal simulations due to some extreme and influential values.

*Precision:* We present mean values of estimated standard errors across simulations, separately for the Taylor series method, SE_*A*_ *(d)*, and the *t*-test (‘*ad hoc*’) method, SE_*B*_ *(d)*. We also present empirical standard errors of the estimated mean differences. For the log-normal simulations, these are calculated as empirical standard deviations over all 10 000 simulations. For the gamma and normal simulations, we present the difference between the 69th and 31st percentiles as an approximately equivalent measure (for a normal distribution, this difference equals the standard deviation).

*Coverage:* Coverage was defined as the percentage of simulations in which a 95 per cent confidence interval, obtained as *d* ± 1.96 × SE*(d)*, included the true difference in means (theoretically or empirically obtained).

Monte Carlo errors for each reported value were calculated, as SD*(d)*/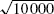 for mean bias, as SD(SE*(d)*)/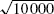 for estimated standard errors, as 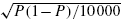 for estimated coverage *P*, and from confidence intervals for medians.

### Results of simulation study

Results for some of the simulations are provided in Tables IV–VI.

**Table VI:** Simulation results for different standard deviations (log-normal distribution); 100 participants in each group; 10 000 simulations in each set.

		Transformation raw to log scale (μ_*z*, 1_, μ_*z*, 2_) and (σ_*z*, 1_, σ_*z*, 2_)				Transformation log to raw scale (μ_*x*, 1_, μ_*x*, 2_) and (σ_*x*, 1_, σ_*x*, 2_)			
			
		Set 13	Set 14	Set 15	Set 16	Set 13	Set 14	Set 15	Set 16
True means (group 1, group 2)		(0, 0)	(0, 0)	(0, 0.2)	(0, 0.2)	(1.65, 1.13)	(1.02, 1.01)	(1.65, 1.38)	(1.02, 1.23)
True SDs (group 1, group 2)		(1, 0.5)	(0.2, 0.1)	(1, 0.5)	(0.2, 0.1)	(2.16, 0.60)	(0.21, 0.10)	(2.16, 0.74)	(0.21, 0.12)
Bias									
Method 1		−0.037	−0.000	−0.037	0.001	−0.008	−0.000	−0.009	0.001
Method 2		−0.366	−0.014	−0.367	−0.014	0.513	0.015	0.565	0.018
Method 3		−0.360	−0.014	−0.366	−0.014	0.513	0.015	0.483	0.015
Standard error									
Method 1	*t*-Test	0.107	0.022	0.107	0.022	0.228	0.023	0.232	0.024
	Taylor	0.284	0.022	0.288	0.022	0.213	0.023	0.218	0.024
	Empirical	0.135	0.022	0.135	0.023	0.215	0.023	0.218	0.024
Method 2	*t*-Test	0.107	0.022	0.107	0.022	0.181	0.023	0.203	0.025
	Taylor	0.125	0.022	0.125	0.022	0.154	0.023	0.172	0.025
	Empirical	0.140	0.023	0.140	0.023	0.154	0.023	0.162	0.024
Method 3	Taylor	0.152	0.023	0.142	0.021	0.112	0.022	0.123	0.025
	Empirical	0.135	0.023	0.138	0.022	0.112	0.022	0.117	0.024
Coverage									
Method 1	*t*-Test	86.5	95.0	86.5	94.4	95.5	95.0	95.8	94.4
	Taylor	98.0	95.0	98.1	94.4	94.5	95.0	94.9	94.4
Method 2	*t*-Test	11.7	89.4	11.0	90.2	17.9	89.4	16.7	90.1
	Taylor	17.5	89.6	16.7	90.4	10.9	89.2	9.8	89.8
Method 3	Taylor	28.3	90.1	21.6	88.6	1.0	89.1	3.4	91.4

Maximum Monte Carlo errors (raw to log) bias: 0.001; *t*-test standard error: 0.0001; Taylor standard error: 0.004; *t*-test coverage: 0.3 per cent; Taylor coverage: 0.5 per cent.

Maximum Monte Carlo errors (log to raw) bias: 0.002; *t*-test standard error: 0.0005; Taylor standard error: 0.0004; *t*-test coverage: 0.4 per cent; Taylor coverage: 0.3 per cent.

*Distributional assumptions met (log-normal distribution):* Table IV. For log-normally distributed data with equal standard deviations (on the log scale) and equal sample size, all methods work well when the standard deviation is small (Table IV, Sets 2 and 4). With a large standard deviation; however, three potential problems are apparent (Table IV, Sets 1 and 3). First, there is bias towards the null in Method 3 for the transformation from the log to the raw scale when the means are not equal. This is because of the omitted third and higher-order terms in the Taylor series. Indeed, we can show that for small difference between the groups and equal standard deviations, Method 3 estimates a fraction 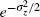 of the true difference. Second, standard errors using the Taylor approximation are inflated when transforming from the raw to the log scale for Method 1. We believe this is because the asymptotic formula requires very large samples to be valid in this case, perhaps because of the large exponential terms. Third, *t*-test-based standard errors are too low for raw to log, and too high for log to raw, with corresponding under- or over-coverage. The conversion is in reality less efficient in the former direction and more efficient in the latter direction than is reflected in these ‘naïve’ standard errors. Empirical standard errors for large standard deviations are larger for Method 1 than for Method 2 converting from log to raw (Table IV, Sets 1 and 3) since in Method 1 the two standard deviations (which are not pooled) are subject to greater variability than the pooled standard deviation in Method 2; empirical standard errors are much smaller for Method 3 from log to raw due to the bias towards the null.

*Small sample sizes: Results not shown; and imbalanced sample sizes*: Table V. Findings were very similar for small sample sizes. The only identifiable sample-size-related problem is an increase in the standard errors for the Taylor approximation method from raw to log, resulting in lower coverage compared with the larger sample size (although in fact producing coverage around 95 per cent for Method 1). When sample sizes are unbalanced, there is bias for large standard deviations in all methods for both transformations (Table V, Sets 9 and 11). This is likely due to a small-sample bias that cancels out across groups when the sample sizes are equal. Coverage for Methods 3, which is adequate when sample sizes are the same, is reduced for unbalanced sample sizes when transforming from raw to log with large standard deviations.

*Different standard deviations:* Table VI. Bias in Methods 2 and 3 (which assume a common standard deviation on the log scale) can be considerable (Table VI, Sets 13 and 15) when the standard deviations are genuinely different. Coverage drops to as low as 1 per cent in one scenario. Method 1 has broadly similar properties to the case of equal standard deviations, although there is a small bias in the point estimate.

*Alternative skew (gamma distribution); and no skew (normal distribution): results not shown*. The transformation from raw to log scales is associated with very little bias. Taylor approximation standard errors are again high for Method 1 when the standard deviation is large. *T* -test-based standard errors are again low for this transformation for both Methods 1 and 2. The transformation from log to raw produces some large biases in all Methods. *T*-test standard errors for this transformation are over-estimated considerably.

Transformations of normally distributed data to the logarithmic scale have good properties in the scenarios simulated. The opposite transformation produced some bias for all three Methods for one scenario with non-zero effect and large standard deviations.

## DISCUSSION

In meta-analysis, it is desirable to combine effects measured on a common scale from as many studies as possible. One obstacle to achieving this is when results are reported on a log-transformed scale for some studies, but on the raw scale for other studies. We have presented several methods for transforming data from two-group studies presented on a logarithmic scale to a raw scale and from a raw scale to a logarithmic scale, thus enabling meta-analyses of all studies to be conducted on one or other scale. The methods also allow a meta-analysis to be undertaken on a log-transformed scale even if all studies report data on the raw scale. This enables estimation of a meta-analytic ratio of geometric means. Such a metric may provide a natural ‘standardization’ across studies, hence reducing heterogeneity, and provides an alternative to the ratio of arithmetic means that is sometimes used [5].

Our first method (Method 1) assumes log-normal distributions with different standard deviations, Method 2 assumes log-normal distributions with a common standard deviation (on the log scale), while Method 3 assumes no particular distribution, but requires similar distributional shapes in the two groups and small effect sizes. On application of the methods to an example, in which most data were reported on the raw scale, we observed some differences between the three methods. Some studies gave substantially different results for Method 1 because of a difference in standard deviations across groups; other studies gave different results for Method 3 because its associated standard errors can be different. In one study, all transformations produced a biased result.

We evaluated the properties of the three methods in a simulation study. This did not reveal a uniformly preferable method. All methods were reasonably robust to data having distributions other than the log-normal. The most serious threat to validity from among the scenarios we simulated was when the standard deviations differed between the groups. Method 1 offers clear advantages in this situation. When standard deviations are large compared with means, biased estimates (in either direction) can be obtained and there is a variation in the precision with which the three methods estimate differences in means: Method 3 produces the most precise estimates when transforming from the log to the raw scale; methods are similar when transforming from the raw to the log scale. We derived a Taylor approximation to the standard error and compared it with a *t*-test-based approach. The Taylor approximation can overestimate standard errors (particularly for raw to log transformations with large standard deviations), but otherwise seems to perform well. The more naïve *t*-test-based approach is less good as it treats transformed means and standard deviations as if they were simple arithmetic means. However, it can be implemented more readily in commonly used meta-analysis software such as RevMan [6], *metan* [7] (for Stata) and Comprehensive Meta-analysis [8]. Its performance is probably adequate for most meta-analytic purposes.

One possible extension to our proposed methods would be to replace our estimators, which are maximum likelihood and therefore may have small-sample bias, with bias-corrected estimators [9]. However, no closed-form standard error is available to our knowledge.

In conclusion, we recommend the use of Method 1 whenever standard deviations are likely to be different in the two groups, with Taylor approximation standard errors for the log to raw transformation. For transformations from raw to log scales, the Taylor approximation standard errors can be large, resulting in down-weighting of these studies in a meta-analysis. When standard deviations are similar, greater precision can be obtained using Method 2, especially when transforming to the raw scale. Method 3 offers a general framework that can be used for different data transformations.

Since the methods allow meta-analyses to be conducted on either the raw or the log-transformed scale, decisions on which scale to use will be required. Several considerations may guide the choice of scale, including (i) fidelity to the data available, by using the scale most frequently reported; (ii) best meeting meta-analytic assumptions, by using the scale believed to have less skew; (iii) minimizing consistency (heterogeneity) of results; (iv) applying the results to another problem (for example, if the results are to feed into a further analysis that requires data on a specific scale). The simulation study did not indicate consistently better properties of one direction of transformation over the other.
